# Multi-Channel MEMS-FAIMS Gas Sensor for VOCs Detection

**DOI:** 10.3390/mi14030608

**Published:** 2023-03-06

**Authors:** Zhujie Zhao, Cheng Lei, Ting Liang, Junna Zhang, Yuqiao Liu, Abdul Ghaffar, Jijun Xiong

**Affiliations:** 1 State Key Laboratory of Dynamic Measurement Technology, North University of China, Taiyuan 030051, China; 2 State Key Laboratory of Geomechanics and Geotechnical Engineering, Institute of Rock and Soil Mechanics, Chinese Academy of Sciences, Wuhan 430071, China; 3 University of Chinese Academy of Sciences, Beijing 100049, China

**Keywords:** gas sensor, MEMS, FAIMS, anodic bonding, VOC gas detection

## Abstract

Aimed at the problems of a large equipment size, long time and high price of environmental VOC gas detection, the FAIMS-VOC gas sensor was designed and prepared according to the principle that the ionization energy of the common VOC gas is less than 10.6 eV. The sensor is small in size, fast in detection, low in power consumption, and can work continuously. The sensor was fabricated through the MEMS process, a specific process which included photolithography, etching, anodic bonding, etc. The sensor is 5160 μm long, 5300 μm wide and 800 μm high. We built a test system to detect two typical VOC gases: isobutylene and acetone. The results show that in the detection of isobutylene gas and acetone gas, the sensor voltage value changes with the change of gas concentration. The linearity of testing isobutylene is 0.961, and the linearity of testing acetone is 0.987. When the isobutylene gas concentration is 50 ppm, the response time is 8 s and the recovery time is 6 s; when the acetone gas concentration is 50 ppm, the response time is 9 s and the recovery time is 10 s. In addition, the sensor demonstrates good repeatability and stability, which are conducive to the detection of VOCs in the environment.

## 1. Introduction

The rapid development of industry has led to increasingly serious environmental problems. The most intuitive manifestation of increased pollution is the decline of air quality [1,2]. In daily life, transportation, housing construction, fuel combustion, etc., may produce volatile organic compounds that endanger human health. VOCs (Volatile organic compounds) have a huge impact on human health. When indoor VOCs reach a certain concentration, they cause headache, nausea and vomiting in a short time. In severe cases, they will damage the brain and nervous system of human body and cause serious consequences such as memory loss [3,4,5]. Outdoor VOCs mainly come from fuel combustion and transportation; indoor VOCs mainly come from the coatings used in home decoration, furniture, cleaners, etc. If there is not enough ventilation to circulate the air and VOCs are present indoors, the indoor air will be much more polluted than the outdoor air [1,2,3,4,5].

Common VOCs are isobutylene, acetone, etc. [6,7,8]. Isobutylene is a volatile organic compound with the chemical formula C_4_H_8_. It is insoluble in water and easily soluble in most organic solvents, such as ethanol. Isobutylene has a stimulating effect on the mucous membranes. The inhalation of high concentrations of isobutylene will produce a suffocating effect, and the maximum allowable concentration is 400 ppm. Isobutylene is 4.5 times more toxic than ethylene. Symptoms caused by the inhalation of isobutylene include: shortness of breath, loss of consciousness and death. In addition, isobutylene can form an explosive mixture when mixed with air, which has the danger of burning and exploding in the presence of a heat source or open flame. Acetone is a highly volatile organic compound with a molecular formula of C_3_H_6_O. It is a colorless and transparent liquid at room temperature and has a pungent odor. It is flammable, volatile and chemically active [9,10]. In industry, it is mainly used as a solvent and is used in plastics, leather, paint, etc.

In industry, various techniques have been used to assess the concentration of VOCs in the air [11,12], such as gas chromatography–mass spectrometry (GC-MS), photoionization detectors (PIDs), and gas-sensitive resistance sensors. Among these techniques, GC-MS is a traditional analysis method [13,14,15,16] which has limitations of a high cost and non-real-time measurement; PIDs, which are usually used in commercial, professional VOC detection systems [17], are easy to carry and have a fast detection speed but are expensive. A gas-sensitive resistance sensor is a type of chemical resistor based on a metal oxide (MO). It has a simple structure [18,19,20,21,22,23,24]; however, it can only detect a specific target gas, so its use is limited. In an isobutylene chemical sensor, Peng-jia Wang et al. synthesized chromium oxide (Cr_2_O_3_) and W-doped Cr_2_O_3_ (W/Cr_2_O_3_) films on the surface of alumina substrate by aerosol-assisted chemical vapor deposition (AACVD). The sensor had good stability, moisture resistance and gas selectivity for isobutylene [25]. In the detection of acetone, Changhui Zhao et al. used Pt-modified CuFe_2_O_4_ nanotubes prepared by electrospinning to detect acetone. The experiment demonstrated good selectivity and long-term stability for acetone. They also used a simple, two-step chemical bath deposition method to prepare an acetone sensor based on α-Fe_2_O_3_/SnO_2_ hybrid nano-array. The test results showed that α-Fe_2_O_3_/SnO_2_ HNAs also demonstrated good reproducibility and selectivity to acetone vapor [26,27].

In this study, we fabricated a vertically structured FAIMS-VOC (Field asymmetric Ion mobility spectrometry-Volatile organic compound) gas sensor for the real-time monitoring of VOC gas. We discussed the working principle of FAIMS, then designed an asymmetric, high-field-strength RF power supply and completed the circuit simulation. We then designed the sensitive chip structure of the gas sensor, developed the related process of chip fabrication and completed the chip fabrication. The FAIMS-VOC gas sensor was tested and analyzed by building a VOC gas test system and using isobutylene and acetone gas.

## 2. Sensor Principle and Sensor Design

### 2.1. Effect of Electric Field on Ion Mobility

Each ion has its own specific ion mobility, and the effect of the electric field on ion mobility is based on the experimental observations of Mason and McDaniel in 1973 [28]. Ion mobility refers to the ratio of the mobility rate of ions in the electric field to the electric field strength [29,30]. Its calculation formula is:

(1) $$K=\frac{V}{E}$$

*K*, *V* and *E* represent the ion mobility, ion mobility rate and electric field strength, respectively.

**Figure 1:**
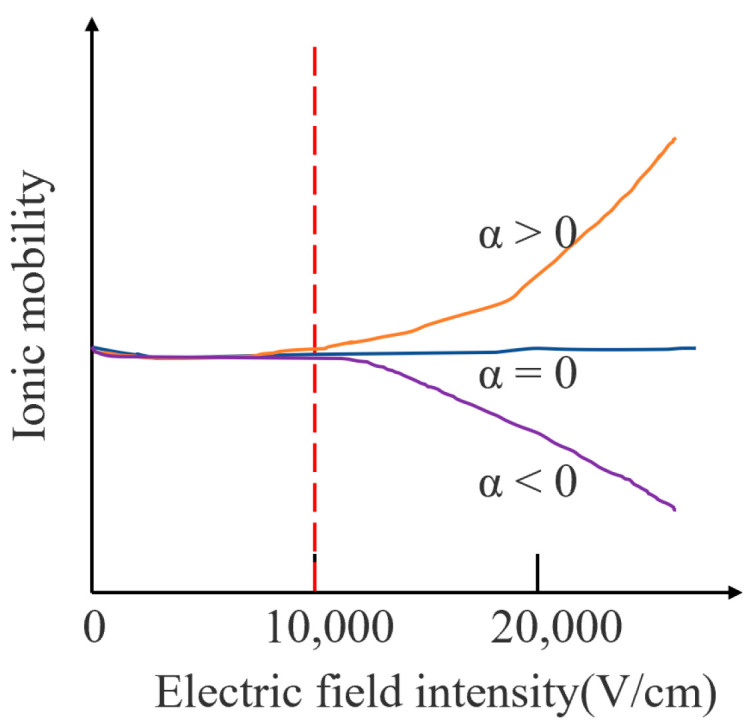
Variation of ion mobility under different electric field conditions.

When the electric field strength is less than 11,000 V/cm, the ion mobility is almost unchanged; therefore, the ion mobility in a low electric field is independent of the electric field strength. When the electric field strength is greater than 11,000 V/cm, the ion mobility coefficient shows a dependence on the electric field strength. Figure 1 shows the change in ion mobility under different electric field conditions. There are three types of ions: the first type is shown by the orange curve in which the ion mobility increases with the increase in the electric field strength; the second type is shown by the blue curve in which the ion mobility does not change with the increase in the electric field strength. The three types are shown on the purple curve. The ion mobility decreases with the increase in the electric field strength, where α is the ion mobility coefficient. Most of the ion mobility changes can be reflected by the above three types.

The mobility coefficient in Figure 1 varies nonlinearly with the electric field strength, which allows ions with different mobilities to be separated at their corresponding high field strength. The expression is shown in (2):

(2) $$K\left(E\right)=K\left(0\right)\left[1+a{\left(E/N\right)}^{2}+b{\left(E/N\right)}^{4}+c{\left(E/N\right)}^{6}+\ldots \right]$$

*K*(0), *K(E)* are the ion mobility under a low electric field and the ion mobility under a high electric field, respectively, N is the concentration of carrier gas *a*, *b* and *c* are the relative relationship between *K*(*E*) and *K*(0) coefficients of deviation, and *E*/*N* represents the number of gas molecules per unit volume.

### 2.2. Circuit Design

**Figure 2:**
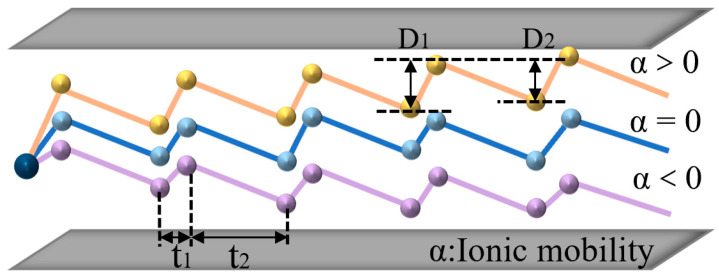
FAIMS ion selection.

When FAIMS works, the ions are moved horizontally by the airflow and the voltage is applied to the plate: a periodic, asymmetric square wave DV and a compensation voltage CV. DV is a periodical, alternating high and low voltage. Due to the different mobilities corresponding to high and low voltages, the ions exhibit a sawtooth trajectory with a small vertical displacement per cycle. Based on this basic theory, if a fixed compensation voltage is superimposed, then the ions that satisfy a specific differential mobility can smoothly fly through the electric field (the difference between the mobility under high field and the mobility under low field; therefore, FAIMS is also called differential ion mobility). Meanwhile, other ions would hit the plate, enabling ion selection, as shown in Figure 2. According to the basic principle of FAIMS, an ideal square wave has extremely fast rise and fall delays, which can effectively improve the resolution of FAIMS. The better the square wave shape, the better the separation performance of the FAIMS. We used the square wave generated by the flyback structure, which can more effectively improve the separation effect of FAIMS [31,32,33,34,35,36,37].

**Figure 3:**
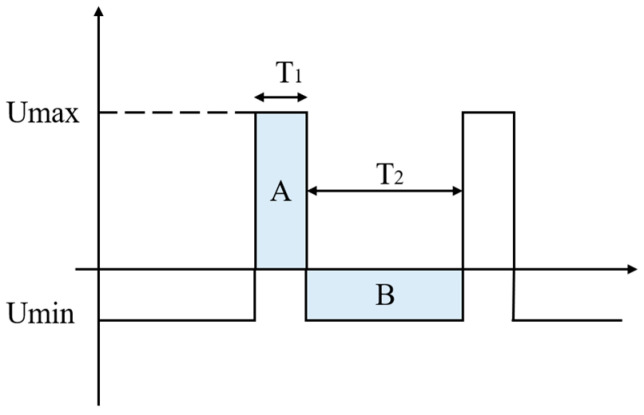
Ideal high-field asymmetric waveform ion mobility spectrometry voltage waveform.

Our target waveform is shown in Figure 3. The electric field strength of the ion in the high field, Umax, is Emax, the motion time is T_1_, and the mobility is K_1_. The electric field strength of the ion in the low field, Umin, is Emin, the movement time is T_2_, and the mobility is K_2_.

To satisfy the condition: E_max_T_1_ = E_min_T_2_ (the areas of the shaded parts A and B are equal: S_A_ = S_B_).

The displacement: D_1_ = K_1_E_max_T_1_, D_2_ = K_2_E_min_T_2_

Assuming K_1_ > K_2_, the ions will move a certain distance in the direction of the high electric field, and the distance moved in a single cycle can be expressed as: ΔD = D_1_ − D_2_.

**Figure 4:**
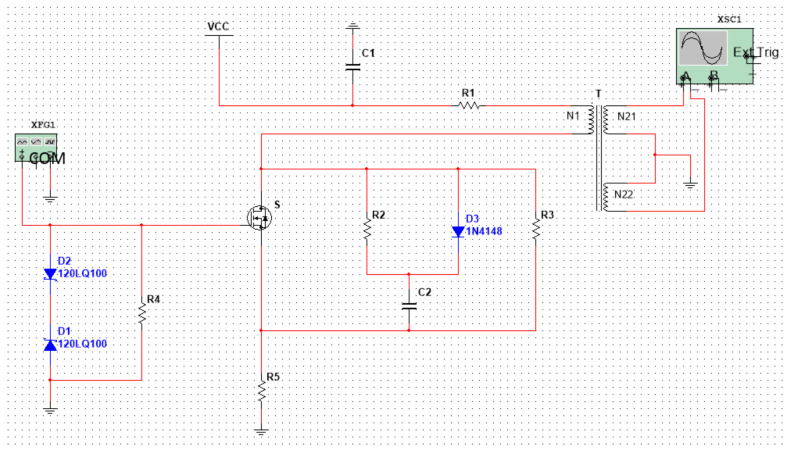
Flyback, high-frequency, high-voltage asymmetric RF electric field.

As shown in Figure 4, in order to obtain a high-frequency asymmetric high field strength and as ideal a square wave as possible, we designed a flyback, high-frequency, high-voltage, asymmetric RF electric field and described and simulated the function of the circuit.

VCC: DC input voltage; T: High frequency transformer; N1: Primary winding; N21, N22: Secondary winding; and S: MOSFET power switch tube. XFG1 is a driving circuit: it generates a signal with an amplitude of 7 V and a frequency of 25 MH, and its function is to quickly turn off and turn on the switch, S.

The working principle of the circuit can be simply described as follows: when the power switch S is turned on, the primary winding N1 generates a current I_1_, and the energy is stored in the primary winding N1. When the power switch tube S is turned off, the primary winding N1 current I_1_ will be disconnected. According to the principle of electromagnetic induction, an induced voltage (also called a reflected voltage) will be generated on the primary winding. At the same time, the secondary windings N21 and N22 generate an induced voltage. As the winding directions of N21 and N22 are opposite to N1, the induced electromotive force generated by the two secondary coils is also opposite to N1, as shown in Figure 4.

**Figure 5:**
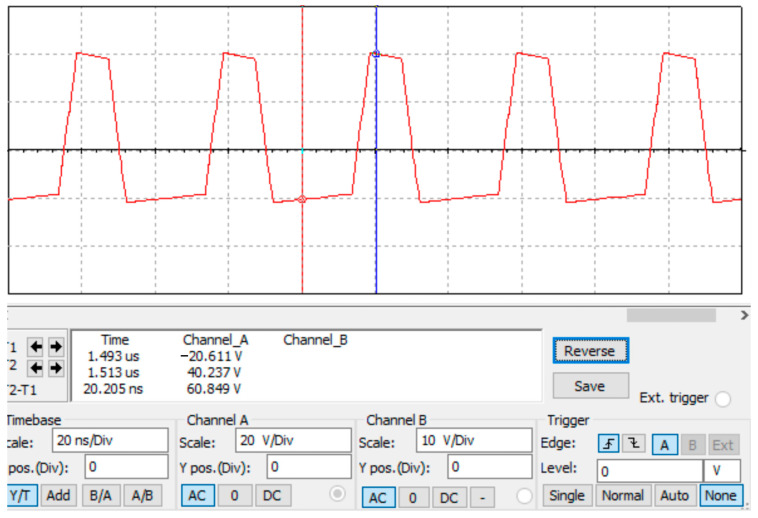
The simulation result of high-field asymmetric waveforms.

After debugging and simulation, we obtained a high-field asymmetric waveform as shown in Figure 5. It can be calculated that a voltage difference of 60 V can generate an electric field strength of 12,000 V/cm in a plate with a spacing of 50 μm. The simulation results satisfy the basic principle of FAIMS.

### 2.3. Manufacturing Steps of the Sensor

**Figure 6:**
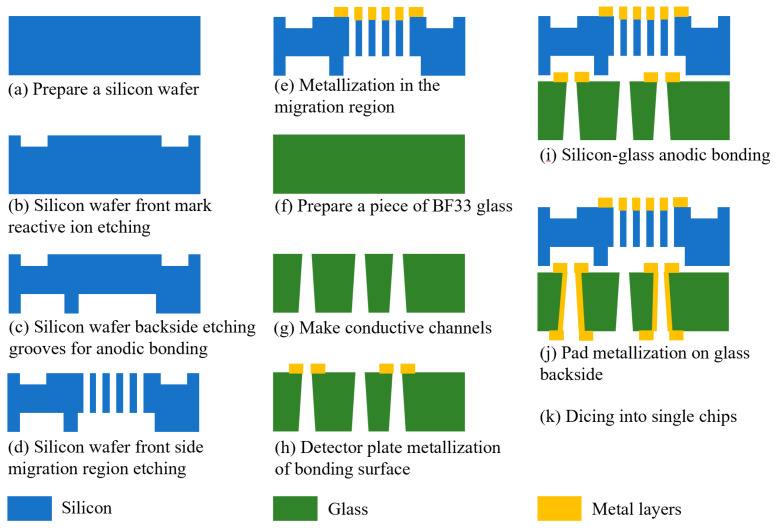
Schematic diagram of each process step in sensor chip fabrication.

When preparing the FAIMS-VOC gas sensor, we chose double-polished silicon wafers ((Suzhou Research Materials Micro/Nano Machining Center) and BF33 glass (Beijing GIN KOO MEMS Scientific & Technological Co., Ltd., Beijing, China) as the chip substrates. The ion migration area was designed and prepared on the glass, and the ion detection area was designed and prepared on the glass. The chip preparation was then completed by anodic bonding. The main process flow is shown in Figure 6: (a) A 4 inch, double-sided, polished silicon wafer with a thickness of 300 µm was selected for the experiment, and the silicon wafer was cleaned according to the RCA standard cleaning method, which is to remove the organic stains and natural stains on the surface of the silicon wafer. (b) The making of the alignment marks: the alignment marks were etched through an RIE process on the front side of the silicon, and the depth of the alignment marks was 500 nm. (c) Grooves for bonding were etched. The DRIE process was performed on the back of the silicon to etch the shallow grooves required for bonding. Their depth was 240 μm. (d) Fabrication of the migration zone: The DRIE process was performed on the front side of the silicon to etch the migration zone channel. Its depth was 60 µm. (e) Metallized migration zone: After process (d) was completed, in order to apply voltage to the channel in the migration region, a layer of metal was sputtered on the migration region on the front of the silicon wafer to make the channel conductive. A Cr/Au metal layer was sputtered by magnetron sputtering coater (EXPLORED, Denton Vacuum Equipment Co., Ltd., Philadelphia, PA, USA). Cr was used as the adhesion layer with a thickness of 50 nm, and Au was used as the electrode layer with a thickness of 500 nm. Cr was chosen as the adhesion layer because it demonstrates a better adhesion to glass than Ti. (f) A 4 inch BF33 glass with a thickness of 500 μm was prepared and cleaned according to the RCA standard cleaning method. (g) Fabrication of conductive vias: through a sandblasting process, circular, frustum-shaped vias were prepared on glass. The diameter of the upper circle was 300 µm, and the diameter of the lower circle was 600 µm. This process was assisted by Beijing GIN KOO MEMS Scientific & Technological Co., Ltd. (h) Preparation of the detection area pole plate: the detection pole plate was prepared on the front side of the glass by a magnetron sputtering process. The pole plate was composed of 50 nm chromium and 500 nm gold. (i) Anodic bonding: the anodic bonding between the silicon wafer and glass was an electrochemical process. The silicon wafer was connected to the anode of the power supply, and the glass was connected to the cathode with a voltage of 800 V. The glass–silicon wafer was heated to 400 °C. Under the action of the voltage, the Na+ in the glass drifted towards the cathode, and a depletion layer formed on the glass surface next to the silicon wafer. The width of the depletion layer was approximately several microns. The depletion layer was negatively charged, and the silicon wafer was positively charged. There was electrostatic attraction between the silicon wafer and the glass, and a thin layer of SiO_2_ was formed at the junction to realize bonding. (j) Making metal pads: the pads on the back of the glass were made through a magnetron sputtering process. The pads were composed of 50 nm chromium and 500 nm gold. (k) Chip cutting: the chips on the wafer were diced into individual chips along the dicing marks, using a dicing machine.

**Figure 7:**
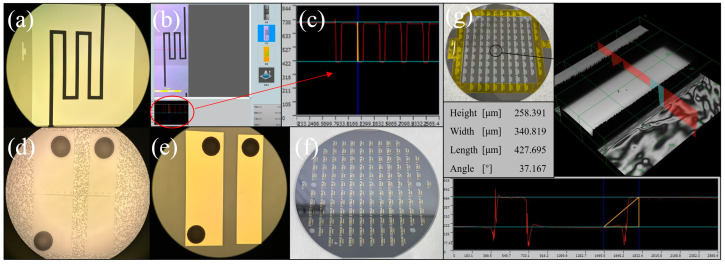
(**a**–**g**) The optical image of MEMS structure.

Figure 7 is a key structural image in the MEMS process. (a) The channel structure in the migration area (b) and (c) are the results of confocal microscope characterization, and the measurement height was 305.34 µm, indicating that the channel etching was successful. (d) is the result diagram of glue spraying, photolithography and the development of the detection electrode on BF33 glass. Considering that Spin Coater can’t make the holes structure on the glass evenly coated with photoresist, the glue spraying process was adopted in the experiment. Observed by optical microscope, it meets the experimental requirements. (e) A detection electrode prepared by the sputtering–stripping process; its appearance is good. (f) The wafer is anodically bonded. It can be seen that there were almost no bonding bubbles except the quasi-marks with a rough surface. The bonding effect is good.

**Figure 8:**
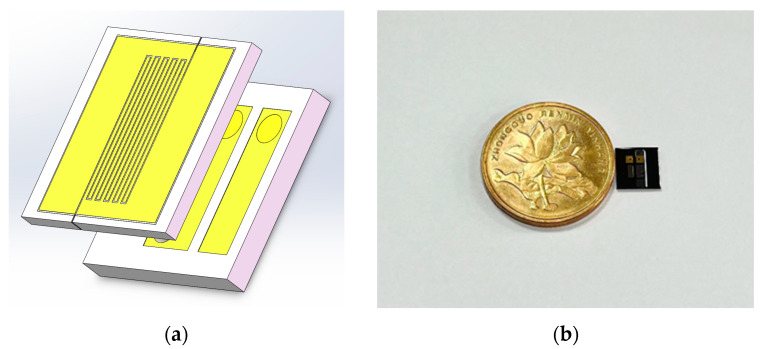
(**a**) Schematic diagram of the 3D structure of the sensor chip; (**b**) comparison of sensor chip and coin size.

Figure 8a shows the structural diagram of the chip, which was mainly composed of the transfer region on silicon and the detection region on glass. The size of a single chip obtained by cutting the wafer prepared in this experiment was 5160 μm long, 5300 μm wide and 800 μm high. Figure 8b is a comparison diagram of a single chip and a pentagonal coin.

## 3. Results and Discussion

### 3.1. VOC Test System

**Figure 9:**
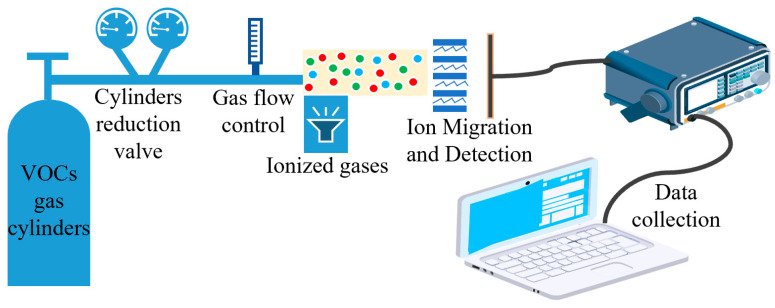
Schematic diagram of the test system.

**Figure 10:**
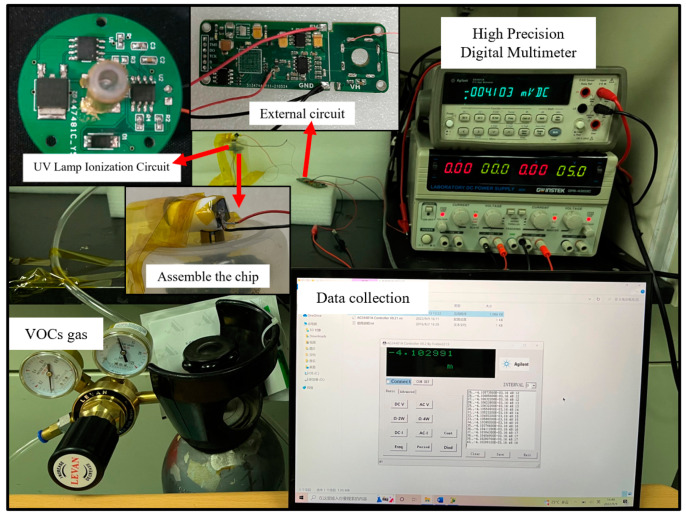
Actual test system.

To test the performance of the sensor, we built a VOC gas test system which consisted of an external VOC gas cylinder, gas ionization zone, gas ion migration zone, gas ion collection zone, and data acquisition system. The VOC standard gases used in the test were all from Taiyuan Tainan Gas Co., Ltd. (Shanxi, China) to ensure reliable gas concentration and experimental safety. We installed pressure-reducing valves and flow controllers for the gas cylinders to ensure a stable gas flow. The gas ionization zone used a photoionization detector. A 10.6 eV UV lamp was used to ionize the particles with an ionization energy below 10.6 eV. The gas ion migration area and the gas ion collection area were prepared by MEMS process, and the gas ion collection area was equipped with a detection circuit to detect the gas ions. The data collection area consisted of high-precision digital multimeters and a PC. The schematic diagram of the test system is shown in Figure 9. The actual test system is shown in Figure 10.

The working principle of the ultraviolet ionization source used in the test system is that the inert gas in the ionization source tube is broken down under the action of high voltage and continues to emit ultraviolet light. Gas collides with the ultraviolet light, and gas molecules with an ionization energy less than the energy of a photon are ionized into gas ions and electrons, as shown in (3):

(3) $$hv+M\to M^{+}+e$$

where *hv* is the high photon energy, *M* is a gaseous sample molecule, *e* is an electron, and *M^+^* is an ionized ion.

### 3.2. Testing Method

In the experiment, the ion recombination during the movement of the migration region was an important reaction to reduce the number of charged particles. Ionized ions may recombine, which would eventually affect the test results. Therefore, the ion recombination in the migration region was analyzed theoretically. Considering that the positive and negative ions in this paper were ionized from the ionization region, it can be assumed that the concentration of positive and negative ions obtained by ionization is always the same, thus simplifying the ion recombination model. The ion recombination equation can be expressed as (4):

(4) $$n=\frac{n_{c}}{1+n_{c}\rho t}$$

where *n_c_* is the initial concentration of ions, the flow rate of the carrier gas was 2 L/min, and the movement time of ions in the migration zone is in the order of ms. Assuming that the ion concentration was 10^7^/cm^3^, the calculated $n_{c}\rho t\ll 1$. Therefore, ion recombination can be ignored when ions move in the migration zone.

We placed the ionization zone and sensor chip in a constant temperature and humidity chamber made of acrylic to ensure the accuracy of the experimental test. Data were collected after stabilization. Considering the experimental safety and testing cost, two VOC gases, isobutylene and acetone, were tested in this experiment. During the test, when the gas concentration and gas type were changed, argon was used to clean the test chamber. Argon is a rare gas that is widely used in industry. Its ionization energy is 15.759 eV, and it will not be ionized. It is very inactive and can neither burn nor support combustion, ensuring the safety and accuracy of experiments.

#### 3.2.1. Isobutylene Test

We choose an isobutylene standard gas with concentrations of 10 ppm, 50 ppm and 100 ppm for testing. The test items included the concentration gradient test, repeatability test and response-recovery test. The gas concentrations corresponding to each cycle in the test experiment are shown in Table 1.

**Figure 11:**
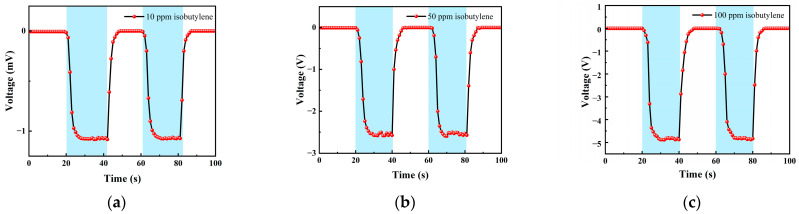
Different isobutylene gas concentration test: (**a**) A 10 ppm isobutylene-Ar cycle test; (**b**) a 50 ppm isobutylene-Ar cycle test; and (**c**) a 100 ppm isobutylene-Ar cycle test.

**Figure 12:**
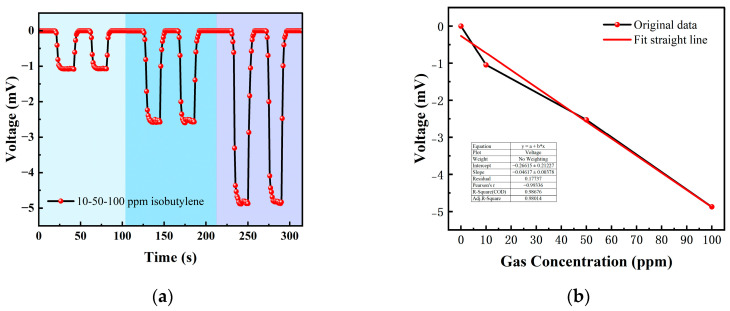
(**a**) 10, 50, 100 ppm isobutylene gas concentration test; (**b**) isobutylene gas concentration–voltage; fitting a straight line.

Table 1 is the gas concentration for each time period. Figure 11 shows the test results of isobutylene gas, and the blue background represents isobutylene. As the gas concentration increased, the signal of the detection plate decreased, indicating that the sensor had a good response to the change in isobutylene concentration. Fitting the stable values measured at each isobutylene concentration, it can be seen from Figure 12b that the FAIMS-VOC gas sensor demonstrates good linearity: the linearity was −0.993.

When the test device was in the atmospheric environment, the average value of the output signal value was −0.006 mV, which could be recorded as the zero point of the sensor; when the isobutylene gas was 10 ppm, the voltage change was 1.051 mV, and the sensitivity at this concentration was 1.051 mV/ppm. In the same way, the sensitivity was 2.526 mV/ppm at 50 ppm, and the sensitivity was 4.876 mV/ppm at 100 ppm. According to the above test results, it can be inferred that the detection accuracy of the sensor for isobutylene can reach 4.876 mV/ppm.

**Figure 13:**
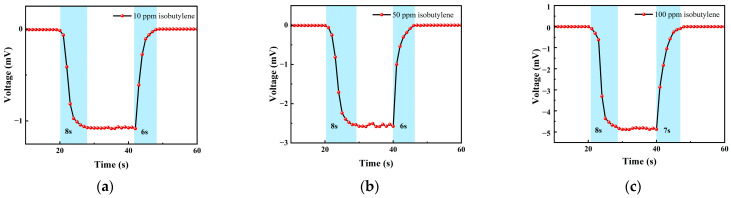
(**a**) A 10 ppm isobutylene gas response–recovery time; (**b**) 50 ppm isobutylene gas response–recovery time; and (**c**) 100 ppm isobutylene gas response–recovery time.

Figure 13 shows the response–recovery time of different concentrations of isobutylene. The analysis shows that when the concentration was 10 ppm, the response time was 8 s and the recovery time was 6 s; when the concentration was 50 ppm, the response time was 8 s and the recovery time was 6 s; when the concentration was 100 ppm, the response time was 8 s and the recovery time was 7 s. It can be seen that the response–recovery speed of the sensor was fast, and the response was consistent under different concentrations.

#### 3.2.2. Acetone Test

We chose acetone standard gas with concentrations of 10 ppm, 50 ppm and 100 ppm for testing. The test items included the concentration gradient test, repeatability test, and response–recovery test. The gas concentrations corresponding to each cycle in the test experiment are shown in Table 2.

**Figure 14:**
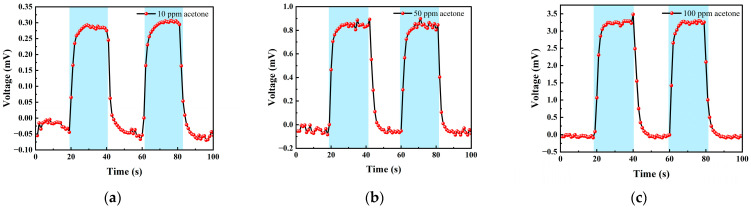
Different acetone gas concentration test: (**a**) 10 ppm acetone-Ar cycle test; (**b**) 50 ppm acetone-Ar cycle test; and (**c**) 100 ppm acetone-Ar cycle test.

**Figure 15:**
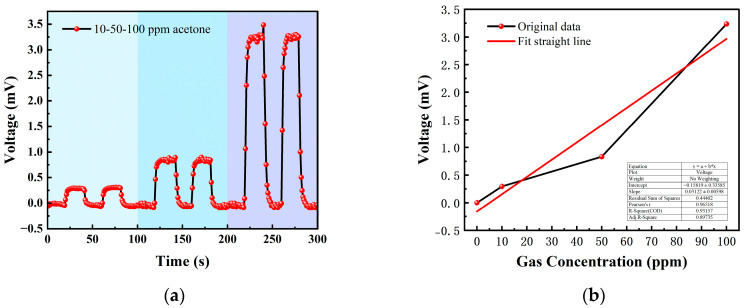
(**a**) 10, 50, 100 ppm acetone gas concentration test; (**b**) acetone gas concentration–voltage fitting straight line.

Table 2 shows the gas concentrations in each time period, and Figure 14 shows the test results of acetone gas, where the blue background represents acetone. As the gas concentration increased, the signal of the detection plate also increased, indicating that the sensor had a good response to the change in acetone gas concentration. Fitting the stable values measured at each acetone concentration, it can be seen from Figure 15b that the FAIMS-VOC gas sensor demonstrated a good linearity for acetone: the linearity is 0.965.

The test device worked in the atmospheric environment, and the average value of the output signal value was −0.055 mV, which could be recorded as the zero point of the sensor; when the acetone gas was 10 ppm, the voltage change was 0.295 mV, and the sensitivity at this concentration was 0.295 mV/ppm. Similarly, the sensitivity was 0.832 mV/ppm at 50 ppm, and the sensitivity was 3.324 mV/ppm at 100 ppm. According to the above results, it can be inferred that the detection accuracy of the sensor for acetone can reach 3.324 mV/ppm.

**Figure 16:**
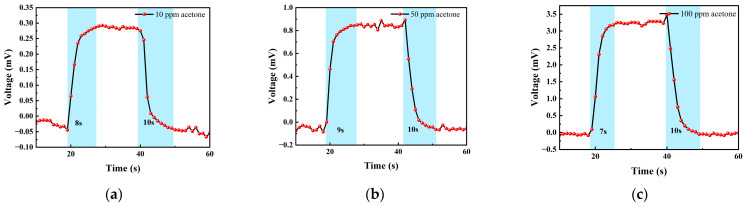
(**a**) 10 ppm acetone gas response–recovery time; (**b**) 50 ppm acetone gas response–recovery time; and (**c**) 100 ppm acetone gas response–recovery time.

Figure 16 shows the response–recovery time of different concentrations of acetone. The analysis shows that when the concentration was 10 ppm, the response time was 8 s and the recovery time was 10 s; when the concentration was 50 ppm, the response time was 9 s and the recovery time was 10 s; when the concentration was 100 ppm, the response time was 7 s and the recovery time was 10 s. As can be seen, the sensor responded slower to acetone than to isobutylene and exhibited poorer stability in the acetone test.

#### 3.2.3. Ar Test

Although the ionization energy of argon is 15.759 eV, it will not be ionized by the UV lamp. However, considering that the test system was placed in an atmospheric environment, argon may carry common gases in the air into the test system, so a separate test for argon was performed. In the experiment, the argon gas was continuously released for 50 s to test the voltage change of the sensor under the argon atmosphere.

**Figure 17:**
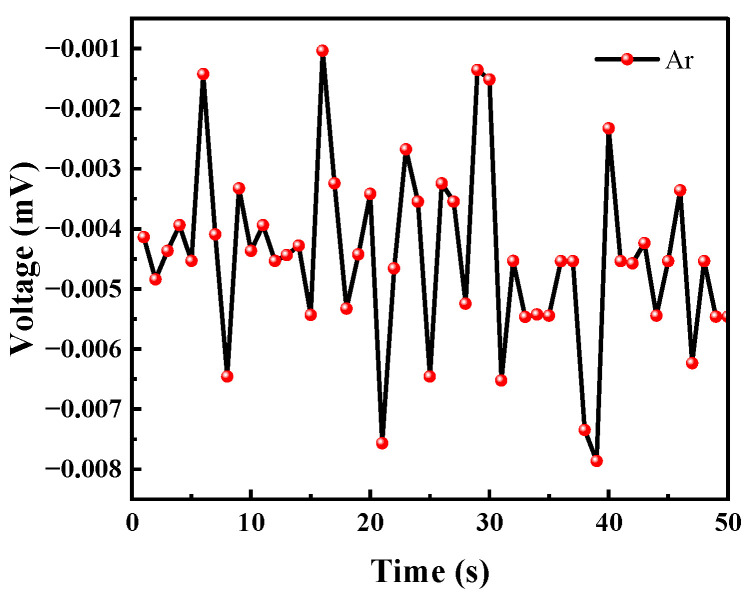
Argon causes voltage changes.

As can be seen from Figure 17, the argon gas caused the voltage value to fluctuate because at the moment when the gas was introduced the balance of the system was broken. This instantaneous pressure change will cause a small change in the voltage value; however, but the voltage change did not exceed 0.01 mV. Therefore, the effect of this phenomenon on the VOC gas detection of the sensor can be ignored.

We also investigated other types of VOC gas sensors published recently and compared them with FAIMS-VOC gas sensors. It can be seen that the sensor in this paper has certain advantages with respect to its detection limit, working temperature and types of VOCs detected. However, the sensitivity needs to be improved.

Table 3 compares the performance of the existing VOC sensor with the FAIMS-VOC sensor in this paper.

## 4. Conclusions

We designed and fabricated a FAIMS-VOC gas sensor chip with multiple migration channels arranged vertically. The chip structure of the gas sensor was fabricated by MEMS technology, and has the advantages of simple processing technology, small size, and easy integration. Considering the experimental safety and cost, the performance of the sensor was verified by testing two typical VOC gases: isobutylene and acetone. According to the test, the sensitivity of isobutylene gas was 4.876 mV/ppm, and the response–recovery time was 8 s and 7 s at 100 ppm. The sensitivity of acetone gas was 3.324 mV/ppm, and the response–recovery time is 7 s and 10 s at 100 ppm. At present, the FAIMS-VOC gas sensor passed the test in the laboratory stage. It is believed that through the optimization and packaging of the sensor, real-time, low-cost and efficient ambient gas detection under more complex conditions can be achieved.

## Figures and Tables

**Table 1:** Gas concentration in isobutylene cycle test.

0–20 s	21–40 s	41–60 s	61–80 s	80–100 s
Ar	10 ppm isobutylene	Ar	10 ppm isobutylene	Ar
Ar	50 ppm isobutylene	Ar	50 ppm isobutylene	Ar
Ar	100 ppm isobutylene	Ar	100 ppm isobutylene	Ar

**Table 2:** Gas concentration in acetone cycle test.

0–20 s	21–40 s	41–60 s	61–80 s	80–100 s
Ar	10 ppm acetone	Ar	10 ppm acetone	Ar
Ar	50 ppm acetone	Ar	50 ppm acetone	Ar
Ar	100 ppm acetone	Ar	100 ppm acetone	Ar

**Table 3:** Comparison of recently published VOC sensors with FAIMS-VOC sensors.

Material	Type	Sensitivity	Detection of Gas Types	Detection Limit (ppm)	Operating Temperature (°C)	References
UV lamp	PID	/	isobutylene	2.2	/	[38]
Si: BF33	FAIMS	1 × 10^−7^/mV	isobutylene	10	RT	[39]
ZnO	Metal oxide	5.71%	acetone	100	200	[40]
GaN	Metal nitride	23%	acetone	500	350	[41]
Si: BF33	FAIMS	4.876 mV/ppm	isobutylene	10	RT	This work
Si: BF33	FAIMS	3.324 mV/ppm	acetone	10	RT	This work

## Data Availability

The data presented in this study are available on request from the corresponding author. The data are not publicly available due to privacy or ethical.
